# Eye problems and musculoskeletal pain in Pokémon Go players

**DOI:** 10.1038/s41598-022-22428-1

**Published:** 2022-11-11

**Authors:** Lukasz D. Kaczmarek, Maciej Behnke, Marzena Dżon

**Affiliations:** Faculty of Psychology and Cognitive Science, Adam Mickiewicz University, 61-664 Poznan, Poland

**Keywords:** Psychology, Health care

## Abstract

Individuals benefit from Pokémon Go (PG) gaming because this mobile augmented reality geolocation video game provides an attractive opportunity to increase physical activity outdoors and socialize. However, based on gaming and electronic media studies, intense involvement with PG is likely related to adverse phenomena, such as arm pain or eye-related problems. We aimed to test how PG use (problematic vs. non-problematic gaming and gaming time) is related to physical symptoms (pain in three body regions and computer vision syndrome). To dissect game-specific effects, we controlled for general problematic smartphone use, phubbing, and electronic media usage. PG players (*N* = 455) completed an online survey. We found that PG players with problematic game use reported more pain and vision problems. Problematic PG use was a better predictor of physical symptoms than PG gaming time and variables related to electronic media use. Problematic PG use and problematic smartphone use were correlated but independent predictors of physical symptoms. We conclude that the type of participation rather than the time spent playing predicts poorer physical health among PG players.

## Introduction

Pokémon Go (PG, Niantic Inc., USA) is an augmented reality geolocation game. PG players explore their surroundings collecting virtual characters called Pokémon. Launched in 2016, PG continues to be one of the most popular and profitable mobile video games, with annual revenue of $904 mln in 2021^1^. Several games with similar mechanics involving geolocation and physical activity have been developed. Some preceded PG, e.g., Ingress (Niantic Inc., USA). Some followed, e.g., *Harry Potter: Wizards Unite* (Niantic Inc., USA) or one of the most recent, *The Witcher: Monster Slayer* (Spokko, Poland).

PG gaming has been related to positive physical, mental, and social outcomes^2–5^. However, little is known about the adverse phenomena among PG gamers, and most of the harmful health evidence is anecdotal. Some PG players can become preoccupied with the game to the extent that they have problems with deliberate control over game use; symptoms indicative of problematic game use^6^. This problematic use, in turn, is likely to increase the risk of pain and vision problems through frequent and prolonged periods of inadequate body posture and bad visual habits^7,8^. We aimed to fill this gap by addressing musculoskeletal pain and vision problems among PG players.

### Pokémon Go versus console and PC video games

PG is an augmented reality mobile game involving physical activity (mostly walking) as a tool for the game's progress. Geolocation video games played with handheld devices differ from console or PC video games. For instance, geolocation games motivate participants to stay outdoors, visit different places, and make real-life friends with other players. This contrasts with stationary console or PC games, where players must remain seated and manipulate the game with a keyboard and mouse or a gamepad. They also minimize gross body movements to maintain greater cognitive focus. Consequently, geolocation games have been identified as offering health and social benefits^6^.

### Pokémon Go versus exergames

Explicit health motives are predictive of PG use^5^. However, an engaging and enjoyable experience rather than physical exercise is the primary motive for endorsing geolocation games^9^. Most PG players exercise while playing regardless of the level of their general physical exercise motivation^9^. Playing PG while not moving is possible because PG involves mechanics enjoyable in a sedentary format, e.g., trading Pokémon with friends, competing with other players online, or using remote gym battles.

Consequently, it is essential to note fundamental differences between PG and exergames (or active video games) that constitute another group of games involving physical activity. Exergames are active video games designed primarily to optimize physical exercise, e.g., in the form of fitness, dancing, boxing, or cycling^10,11^. Exergames have an explicit health or fitness focus. Moreover, exergames are often carefully designed to augment the treatment of patient populations^12^.

Geolocation augmented reality games might be considered a midpoint between stationary video games and exergames in required physical activity. Researchers and practitioners should be careful in generalizing the health outcomes of games that involve minimal physical activity (e.g., PC games), involve instrumental use of physical activity (e.g., geolocation games such as PG) from games where physical activity is the developers' and players' primary concern.

### Pokémon Go and problematic video games use

Some individuals develop an intense interest in video games resulting in problematic gaming^13–15^. Problematic gaming presents a compulsive use of video games that negatively influences gamers' lives^16^. Problematic gaming affects up to 8.5% of gamers^17,18^. It is an individual and public health problem that predicts lower educational achievements^17–19^, attention deficits^20^, aggressive behavior, depression, anxiety^21^, and deficits in real-life relationships^22^. The primary problematic gaming mechanism is related to built-in reward systems with repetitive conditional rewards that facilitate intensely pleasurable experiences through increased dopamine release^23,24^. This mechanism is similar to behavioral addictions, such as gambling^14^.

Although PG's beneficial aspects are often emphasized, PG's design and monetization scheme share adverse psychological effects of other free-to-play mobile games. For instance, PG has developed a system of rewards with gambling mechanisms, i.e., players wager their resources (e.g., time and effort) on events with an uncertain outcome expecting to obtain a payoff. The core of PG is the wild Pokémon hunt. Individuals walk around their neighborhood expecting to find desired Pokémon (e.g., rare species with a low probability of spawning or specimens missing in their collection). Players can intensify the collecting process by purchasing items (e.g., lure modules or egg incubators) with real-world money that provides more opportunities to draw a Pokémon. Finding and catching a rare Pokémon is a rewarding experience that motivates the continuation of behavior by fueling the excitement related to the anticipation of future rewards. Such mechanisms in which individuals invest their time, physical effort, or money with an expectation of in-game prizes (e.g., unique Pokémon) copy a psychological mechanism typical for slot machines, roulette wheels, or capsule toy vending machines^23^. Several other possible mechanisms increase the potential of PG for problematic gaming. For instance, PG can be played every day and all day^25^, and players are rewarded for regular daily use^26,27^.

### PG gaming and physical health

PG players are more likely to maintain inadequate body posture related to heavy electronic device use, termed iPosture, Nintendo neck, or tech neck^7,28^. Consequently, structural changes are likely to occur and produce chronic headaches and neck and shoulder pain^29–31^. Highly engaged PG players walk a lot in the bowed-over position, with arms bent in their elbows and stretched wrists to hold their smartphones. Similar behaviors related to prolonged handheld media use were related to adverse health effects in back pain^32^ or hand deformation^33^. However, the evidence of these phenomena is often preliminary or anecdotal.

PG players spend too much time in front of screens^34,35^. For these reasons, intense PG gaming is likely to lead to vision problems such as computer vision syndrome (digital eye strain or visual fatigue) related to accommodation (e.g., difficulty refocusing from one distance to another) and to dry eye (e.g., irritated/burning eyes)^8^. Computer vision syndrome affects most heavy computer users^36^.

There has been only one study that examined physical symptoms among PG players^37^. That study reported no differences between players and non-players. However, that study had some limitations because it used a scale that mixed symptoms that were likely to be related to mobile gaming (e.g., neck pain) with symptoms that were not likely to be affected by video gaming (e.g., constipation and diarrhea).

### Pokémon Go gaming, phubbing, and problematic smartphone use

Adverse health effects of problematic PG gaming operate with other problematic behaviors related to the heavy use of mobile phones, such as problematic smartphone use^38^ and phubbing^39^. Problematic smartphone use is a psychological or behavioral dependence on smartphone-based activities. It distracts users from their daily lives and undermines their self-regulation capacity^38^.

Furthermore, phubbing (a combination of the words “phone” and “snubbing”) occurs in social situations when individuals maintain focus on their mobile phones at the expense of the interlocutor, who may feel ignored or snubbed^40,41^. Phubbing might be related to physical symptoms because it indicates that an individual’s dependence on a smartphone extends to social situations. Consequently, even social situations (e.g., a party or meeting a friend in a cafe) do not distract individuals prone to phubbing from their mobile phones. They do not serve as motives to limit electronic screen exposure and adopt a healthier posture. These more fundamental aspects of smartphone use are essential to control the effects specific to PG gaming.

Finally, the motives for which players play (e.g., intense preoccupation with the game and self-control difficulties among problematic gamers) might result in more severe physical outcomes than more extended periods of recreational gaming. Namely, gaming time may predict physical symptoms less than the high intensity of problematic gaming. For instance, problematic PG players might focus entirely and uninterruptedly on the game and the smartphone. In contrast, non-problematic PG players are more likely to be distracted from the game and attracted by the surroundings or other brief activity opportunities. Consequently, non-problematic PG players might take breaks more often during one gaming session. They can defocus from the game to appreciate places, nature, weather, or interact with passer-byes. This is likely to make a difference. Ophthalmologists recommend breaks as short as 20 s, focusing eyes on an object 20 feet away every 20 min to relieve the eyes and reduce the risk of computer vision syndrome^42^.

### Gaming time and problematic game use

There is converging evidence that problematic video gaming rather than time spent on video games predicts adverse outcomes. For instance, players' engagement in problematic gaming is more predictive of health (including physical health evaluation and somatic health problems) than heavy use over time^43^. More robust findings extend to mental health and life achievements. For instance, problematic gaming is related to clinical problems, but gaming time is not^44^. Another study revealed that gaming time is not predictive of adverse effects compared to problematic game use, which is a moderate predictor^45^. Moreover, studies suggest that gaming time might predict positive outcomes (e.g., more support from other players) after controlling for negative gaming motivations^46^.

### Present study

We aimed to address the health risks related to PG gaming, emphasizing the problematic PG gaming manifested in gaming behavior control difficulties. We accounted for problematic gaming (e.g., gaming due to compulsive urges) and gaming time (how much players play). This distinguishes between players who engage with a problematic approach and those who do not present a problematic use. We also aimed to examine whether PG-specific problematic use explains more than the more general phenomena related to problematic smartphone use. We expected that problematic PG gaming and, to a lesser extent, PG gaming time would be related to physical health problems, i.e., pain and computer vision syndrome.

## Methods

### Participants

The participants were 455 PG players (43.9% women) aged 18 to 53 years (*M* = 26.45, *SD* = 6.17). On average, participants used electronic media for 47.56 h per week (*SD* = 29.92), nearly a quarter of which was spent playing PG (*M* = 11.79 h per week, *SD* = 10.09). A power analysis indicated that to detect the expected small effect sizes B = 0.20, with a power of 0.80, at least 444 participants would be required^47^. We used Facebook ads targeted at PG players. Participants did not receive any compensation for their participation. The study was in accordance with the Declaration of Helsinki. The requirement for ethical approval was waived by institutional Ethics Committee in line with National Science Center guideliness. All participants were informed about the study, and all provided signed informed consent.

### Measures

#### Problematic PG use

To measure problematic PG use, we adapted eight items from the Game Addiction Scale and the Internet Addiction Scale^48^, accounting for preoccupation with PG ("I dream about the times to play Pokémon Go when I don't play the game"), PG withdrawal ("I feel anxious when I don't play Pokémon Go"), self-control difficulties ("I cannot stop playing Pokémon Go, although I want to"), and the negative influence of PG gaming on daily life ("I neglect my duties to be able to play Pokémon Go"). We adjusted the original items so that they fit the PG context. Participants responded on a 7-point scale ranging from "I strongly disagree" to "I strongly agree." The scale had satisfactory internal consistency (α = 0.81).

#### Problematic smartphone use

We used a 7-item Mobile Phone Use Addiction Scale^48^ to quantify problematic smartphone use (e.g., "I never turn off my mobile phone," "I cannot think of a life without my mobile phone," I had times when I forgot to do things since I was very busy with the mobile phone"). Participants answered on a 7-point scale ranging from "I strongly disagree" to "I strongly agree." The scale had satisfactory internal consistency (α = 0.78).

#### Phubbing

We used a 4-item Phubbing Scale^48^ to measure the problematic use of a smartphone extending to social situations (e.g., ”My eyes start wandering on my phone when I'm together with others.,” ”People complain about me dealing with my mobile phone.”, “I'm busy with my mobile phone when I'm with friends.”). Participants answered on a 7-point scale ranging from "I strongly disagree" to "I strongly agree." The scale had satisfactory internal consistency (α = 0.88).

#### Visual symptoms

Participants reported computer vision syndrome using subscales from a validated instrument with symptoms related to heavy computer use^49^. We asked, "To what extent do you experience…?". Participants reported eye accommodation problems with four items (blurry vision at near, intermediate, and far distances and difficulty or slowness in refocusing eyes from one distance to another) and eye strain with five items (irritated or burning eyes, dry eyes, eye strain, headache, and sensitivity to bright lights). Participants estimated the severity of visual symptoms using a scale ranging from 0 "none" to 6 "severe." The reliability was α = 0.80 for accommodation problems and α = 0.85 for eye strain.

#### Musculoskeletal symptoms

Participants reported discomfort in musculoskeletal symptoms by responding to a question, "To what extent do you experience discomfort in your…?"^49^. Three items asked about the upper limbs (fingers, hand/wrist, elbow/forearm), four items about the back (neck, shoulder, upper back, and lower back), and three items about the lower limbs (ankle/foot, lower leg, thighs/knees)^49^. The validity of brief rating scales of physical symptoms has been supported by comparing subjective ratings with clinical evaluations^50^. Participants used a scale ranging from 0 "none" to 6 "severe." The internal consistency of the scales was α = 0.76 for the upper limbs, α = 0.59 for lower limbs, and α = 0.79 for the back.

#### Pokémon Go gaming time

Participants reported how much they played PG^5,51,52^. First, they were asked, "How many days have you played Pokémon Go in the last 7 days?" and responded on an 8-point scale ranging from (0—"I did not play" to 7 "every day"). They reported the usual daily time answering a question, "How much time on one day did you generally play Pokémon Go over the past 7 days?". They reported gaming hours and/or minutes. We calculated the total gaming time by multiplying the number of days by daily minutes.

#### Media usage time

Participants reported their media use time by answering a question, "How many days did you use a TV, desktop computer, game console, laptop, tablet, smartphone, etc., in the last 7 days? This includes work and leisure". They used an 8-point scale ranging from (0—"never" to 7 "every day"). We measured the daily media use time with a question, "How much time on one day did you generally use a TV, desktop computer, game console, laptop, tablet, smartphone, etc., in the past 7 days. This includes work and leisure." We calculated the media usage time by multiplying the number of days by daily minutes.

### Statistical analysis

We performed path analysis to test whether physical symptoms were predicted by problematic PG gaming, problematic smartphone use, and phubbing after controlling for gaming time, electronic media exposure, age, and gender. To address multicollinearity between predictors, we calculated variance inflation factor (VIF), with values < 5.00, and Tolerance (TOL), with values > 0.20, indicating an acceptable level of multicollinearity between variables. We evaluated the model fit with the Root Mean Square Error of Approximation (RMSEA; values < 0.06 indicating a good fit^53^) and Comparative Fit Index (CFI; values > 0.90 indicating a good fit^53^). The maximum likelihood estimator with Satorra-Bentler adjustment (MLM) was used with mPlus 8.5^54^. Outliers (above 3.29 standard deviation) were excluded^55^.

## Results

**Figure 1:**
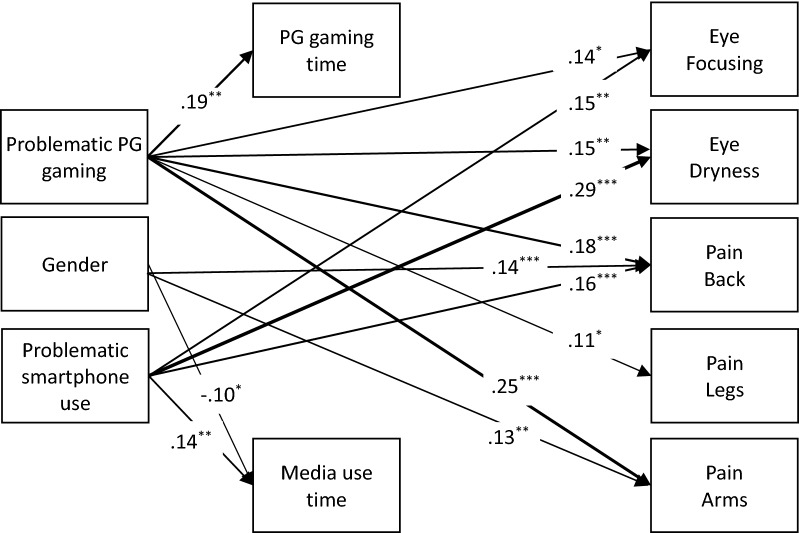
Physical symptoms among Pokémon Go players. The figure presents standardized beta coefficients between variables. Phubbing and age were unrelated to other variables and are omitted from presentation. PG = Pokémon Go. Gender coded as 0 = men, 1 = women. ^*^p < .05, ^**^p < .01, ^***^p < .001.

Table 1 presents descriptive statistics and intercorrelations. There were no problems of multicollinearity between predictors with all VIF’s < 1.63, and TOL values > 0.61. The model (Fig. 1) fit the empirical data well, χ^2^ (38) = 66.89, *p* < 0.01, RMSEA = 0.04, 90% CI [0.02, 0.06], CFI = 0.95. Insignificant paths had no effect on the model fit, Δχ^2^ (32) = 42.41, *p* = 0.10.

**Table 1:** Descriptive statistics and correlations among study variables.

	*M*	*SD*	1	2	3	4	5	6	7	8	9	10	11
1. Problematic PG gaming	16.64	8.22											
2. Problematic smartphone use	27.47	8.67	.38**										
3. Phubbing	12.62	5.26	.24**	.52**									
4. Eye focusing	3.13	4.31	.19**	.20**	.17**								
5. Eye dryness	7.34	6.48	.26**	.34**	.25**	.46**							
6. Pain back	5.30	5.05	.23**	.23**	.16**	.34**	.50**						
7. Pain arms	2.08	2.92	.23**	.08	.11*	.30**	.32**	.47**					
8. Pain legs	1.58	2.53	.11*	.05	.08	.18**	.38**	.37**	.40**				
9. PG gaming time [h/week ]	11.79	10.09	.18**	− .01	.02	− .04	− .05	− .07	.01	− .06			
10. Media use time [h/week]	47.56	29.92	− .02	.12*	.02	.02	.10*	.06	.04	.07	.10*		
11. Age [yrs]	26.45	6.17	− .05	− .16**	− .03	.05	− .03	.07	.05	.07	.06	.03	
12. Sex	–	–	− .01	.06	− .04	− .06	− .05	− .07	.02	− .06	.06	− .11*	.04

The scale of all scores ranged for Problematic PG gaming 8–56, Problematic smartphone use 7–49, Phubbing 4–28, Eye focusing 0–24, Eye dryness 0–30, Pain back 0–24, Pain arms 0–30, Pain legs 0–18. Sex coded as 0 = men, 1 = women. PG gaming time and media use time are reported in hours per week.

*PG* Pokémon Go.

*p < .05. **p < .01.

Supporting our hypothesis, PG players with higher levels of problematic PG gaming reported more severe symptoms regarding vision (eye focus and eye dryness) and pain (back, legs, and arms) than players low on problematic PG gaming. While controlling for problematic PG gaming, time spent playing PG was not predictive of physical symptoms.

## Discussion

We examined physical symptoms among PG players and their relationship with PG gaming time and PG use ranging from non-problematic to problematic. We found that PG players with more problematic gaming levels (but not longer gaming time) were more likely to exhibit physical symptoms. PG-related variables predicted the outcomes above the effects of problematic smartphone use and broad exposure to electronic media. Our findings contribute to the literature by documenting adverse effects among problematic PG gamers that could be considered to determine PG gamers' overall health risks and benefits.

We found that problematic PG gaming was moderately related to problematic smartphone use. This indicates that some PG players have a general problem with controlled smartphone use. Intense participation in PG gaming provides strong motivation for smartphone preoccupation and compulsive use. However, many problematic PG players have no problems with smartphone overuse. Further studies might use prospective or experimental study designs to determine to what extent problematic PG gaming leads to problematic smartphone use and to what extent individuals with problematic smartphone use are more likely to get hooked on PG. Such designs would also allow testing indirect effects (e.g., whether problematic PG gaming leads to more prolonged PG gaming and then to physical outcomes) rather than testing gaming type and time as concurrent predictors. Studies might be extended to children who might be initially attracted to smartphones by the Pokémon franchise and develop general smartphone problematic use starting with this type of gaming experience. Finally, problematic PG gaming was weakly related to phubbing. This suggests that the gaming activity of PG players weakly interferes with their social relationships^40,41^. It might indicate that phubbing is less relevant to PG players because their use of mobile phones during social interaction is more acceptable due to the augmented reality game conventions.

Problematic PG gaming was substantially predictive of physical complaints' severity. Problematic PG gaming was related to greater vision problems and pain. It is noteworthy that problematic smartphone use predicted only back pain. Problematic PG gaming also predicted more pain in the legs and arms—the parts of the body characteristic of games that require walking while holding smartphones in a flexed hand with an arm bent. Problematic PG players also complained on computer vision syndrome, i.e., dry eye and problems with efficient eye focusing that resulted in decreased visual acuity. These findings review a common belief among problematic PG players who expect more benefits from PG gaming compared to casual users^56^. Evidence of the adverse effects of PG gaming might serve as a persuasive argument to promote health for PG players—especially those at risk of developing problematic PG use, i.e., strongly motivated and passionate about playing PG^57–59^.

Finally, we replicated previous findings that gaming time is not predictive or is a minor predictor of adverse outcomes after controlling for problematic game use^43–46^. This indicates that players might play longer if only they maintain control over their gaming behavior. Further studies might test whether gaming behavior patterns (e.g., taking brief breaks, stretching, or refocusing on the surroundings) differ between problematic and non-problematic PG gamers during extended gaming periods.

### Practical implications

Our study presents a negative facet of a particular augmented reality geolocation mobile game's use. Thus, our results add to the ongoing debate on video games as a method of facilitating health. General conclusions are difficult to formulate because researchers, practitioners, and game developers are becoming increasingly aware of games' diversity and specificity in their aims and primary (intentional) and secondary (unintentional) outcomes. Several authors and organizations excluded specific types of games (i.e., active video games) as an optimal method of promoting regular and intense physical activity^60,61^. Others emphasize the accumulation of positive evidence regarding specific types of games, e.g., fitness or boxing games^10,11^.

Moreover, our work indicates the benefits of an extended range of health outcomes examination in balancing video games' pros and cons. This balanced perspective is important because some previous reviews focused on PG advantages' neglecting possible adverse effects^2^. Focusing exclusively on the bright or dark side of video gaming makes it problematic to determine games' total health effects. Our study serves as an example of how the game's primary effects identified in previous studies (i.e., walking more and spending more time outdoors)^2,3,5^ are accompanied by secondary risk factors inherent to the game’s technology. It is essential to focus on both groups of effects, as previous studies on PG almost uniformly advocated for its benefits suggesting that PG players might be healthier than non-players, and PG players who play more might be healthier than those who play less^3,5^. In contrast, our study indicates that problematic players suffer from physical problems typical of electronic media overuse, i.e., eye problems and musculoskeletal pain typical for handheld devices’ overuse. PG players might benefit from increasing their awareness of computer vision problems and iPosture pain prevention. For instance, they might monitor and regulate their posture to relieve the intense pressure on the spine. Although similar recommendations have long been formulated regarding smartphone use^62^, extending these recommendations to PG players is imperative.

### Limitations and future studies

This study had several limitations. First, our research design was cross-sectional. Longitudinal designs would provide direct evidence of causality. Although regressive models allow for the interpretation of causal effects^63^, experimental designs that manipulate gaming behaviors offer direct evidence of causality. Further studies might test whether problematic PG gaming reduction interventions reduce physical symptoms^64,65^. Second, we focused on novel outcomes resigning from measuring PG players' physical activity as a well-established variable^2,3,5^. Future studies might account for gaming time, physical activity levels, and sitting time to directly link the symptoms with specific aspects of physical activity. Third, the reliability of leg pain measurement was below the conventional threshold. Therefore, results related to the legs should be interpreted with caution. Fourth, we did not measure whether the pain reported by our participants was acute or chronic. It might be worthwhile distinguishing these two pain forms due to concerns that Pokémon Go players might be more prone to injury^66^. Fifth, we adjusted the scales for problematic PG use, problematic smartphone use, and phubbing to the context of PG gaming. This reduces the feasibility of comparing our conclusions with the results of other studies. Sixth, problematic PG use, problematic smartphone use, and phubbing asked about subjective criteria, whereas the physical outcomes asked about objective outcomes. Seventh, we recruited participants using Facebook ads targeted at PG players. Our strategy might have excluded individuals that were not active users of these social media platforms. Future studies may use diverse recruiting strategies, e.g. beyond social media ads, reaching PG players of all ages and digital fluency levels to generalize our conclusions. Finally, pain self-reports and clinical evaluation are highly correlated^50^. However, more substantial support for our hypothesis would result from health problems documented with objective methods.

## Conclusions

We report novel findings that problematic PG gaming is related to physical health problems. This is in line with evidence that PG gaming styles and intensities determine PG gaming costs and benefits. These findings are essential since PG is often presented as promoting healthy activity during leisure. On the contrary, we emphasize that problematic PG users are at an increased risk of physical health problems. Understanding the possible adverse effects and correlates of PG gaming is vital to developing engaging games that facilitate health behaviors (e.g., outdoors walking) and minimize the potential harm to other aspects of physical health.

## Data Availability

Study’s data and syntax available at: https://osf.io/nasdy/.
